# Ultrahigh-field cardiovascular magnetic resonance T1 and T2 mapping for the assessment of anthracycline-induced cardiotoxicity in rat models: validation against histopathologic changes

**DOI:** 10.1186/s12968-021-00767-8

**Published:** 2021-06-17

**Authors:** Heae Surng Park, Yoo Jin Hong, Kyunghwa Han, Pan Ki Kim, Eunkyung An, Ji Yeon Lee, Chul Hwan Park, Hye-Jeong Lee, Jin Hur, Young Jin Kim, Byoung Wook Choi

**Affiliations:** 1 Department of Pathology, Ewha Womans University Seoul Hospital, 260 Gonghang-daero, Gangseo-gu, Seoul, 07804 Republic of Korea; 2 Department of Radiology and Research Institute of Radiological Science, Yonsei University College of Medicine, Severance Hospital, 50-1 Yonsei-ro, Seodaemun-gu, Seoul, 03722 Republic of Korea; 3 Department of Radiology and Research Institute of Radiological Science, Yonsei University College of Medicine, Gangnam Severance Hospital, 211 Eonjuro, Gangnam-gu, Seoul, 06273 Republic of Korea

**Keywords:** Magnetic resonance imaging, Myocardial fibrosis, Edema, Inflammation, T1 mapping, Cardiotoxicity, Rats

## Abstract

**Background:**

Chemotherapy-induced cardiotoxicity is a well-recognized adverse effect of chemotherapy. Quantitative T1-mapping cardiovascular magnetic resonance (CMR) is useful for detecting subclinical myocardial changes in anthracycline-induced cardiotoxicity. The aim of the present study was to histopathologically validate the T1 and T2 mapping parameters for the evaluation of diffuse myocardial changes in rat models of cardiotoxicity.

**Methods:**

Rat models of cardiotoxicity were generated by injecting rats with doxorubicin (1 mg/kg, twice a week). CMR was performed with a 9.4 T ultrahigh-field scanner using cine, pre-T1, post-T1 and T2 mapping sequences to evaluate the left ventricular ejection fraction (LVEF), native T1, T2, and extracellular volume fraction (ECV). Histopathological examinations were performed and the association of histopathological changes with CMR parameters was assessed.

**Results:**

Five control rats and 36 doxorubicin-treated rats were included and classified into treatment periods. In the doxorubicin-treated rats, the LVEF significantly decreased after 12 weeks of treatment (control vs. 12-week treated: 73 ± 4% vs. 59 ± 9%, *P* = 0.01).  Increased native T1 and ECV were observed after 6 weeks of treatment (control vs. 6-week treated: 1148 ± 58 ms, 14.3 ± 1% vs. 1320 ± 56 ms, 20.3 ± 3%; *P* = 0.005, < 0.05, respectively). T2 values also increased by six weeks of treatment (control vs. 6-week treated: 16.3 ± 2 ms vs. 10.3 ± 1 ms, *P* < 0.05). The main histopathological features were myocardial injury, interstitial fibrosis, inflammation, and edema. The mean vacuolar change (%), fibrosis (%), and inflammation score were significantly higher in 6-week treated rats than in the controls (*P* = 0.03, 0.03, 0.02, respectively). In the univariable analysis, vacuolar change showed the highest correlation with native T1 value (R = 0.60, *P* < 0.001), and fibrosis showed the highest correlation with ECV value (R = 0.78, *P* < 0.001). In the multiple linear regression analysis model, vacuolar change was a significant factor for change in native T1 (*P* = 0.01), and vacuolar change and fibrosis were significant factors for change in ECV (*P* = 0.006, *P* < 0.001, respectively) by adding other histopathological parameters (i.e., inflammation and edema scores)

**Conclusions:**

Quantitative T1 and T2 mapping CMR is a useful non-invasive tool reflecting subclinical histopathological changes in anthracycline-induced cardiotoxicity.

**Supplementary Information:**

The online version contains supplementary material available at 10.1186/s12968-021-00767-8.

## Background

Chemotherapy-induced cardiotoxicity is a well-recognized adverse effect of chemotherapy. Anthracyclines are the representative agents that cause cardiotoxicity in a dose-dependent manner. Many mechanisms of anthracycline cardiotoxicity have been suggested, but the main mechanism is thought to involve the iron-dependent generation of reactive oxygen species and subsequent widespread oxidative damage to cardiomyocyte [1]. This results in myofibrillar loss and cellular necrosis and can lead to irreversible diffuse myocardial fibrosis [2, 3], which is known to be associated with adverse cardiac events [4]. Since cardiotoxicity causes irreversible cardiac damage, early diagnosis and treatment are clinically important [5].

Measurement of left ventricular (LV) ejection fraction (LVEF) is standard practice for cardiotoxicity monitoring during cancer therapy [6, 7]. However, a previous study found that the myocardial tissue and cardiovascular magnetic resonance (CMR) parameters change before contractile dysfunction and irreversible changes occur [8, 9]. Other studies also demonstrated that subclinical myocyte injury occurs at lower and intermediate cumulative doses with preserved LV systolic function [10–12].

Previous studies have identified histological findings associated with this condition, such as myocardial fibrosis and myocyte edema and injury [8, 13–15]. CMR could quantitatively evaluate some tissue changes in animal models of cardiotoxicity [8, 14]. Thus, in the present study, we aimed to evaluate histopathologic changes associated with anthracycline-induced cardiotoxicity in rat models more comprehensively, and validate the native T1, extracellular volume (ECV), and T2 values acquired using ultrahigh-field T1 mapping CMR against histopathologic features. In addition, we aimed to compare native T1, T2, ECV, and histopathologic findings across treatment periods.

## Material and methods

### Experimental design and sample size calculation

**Fig. 1:**
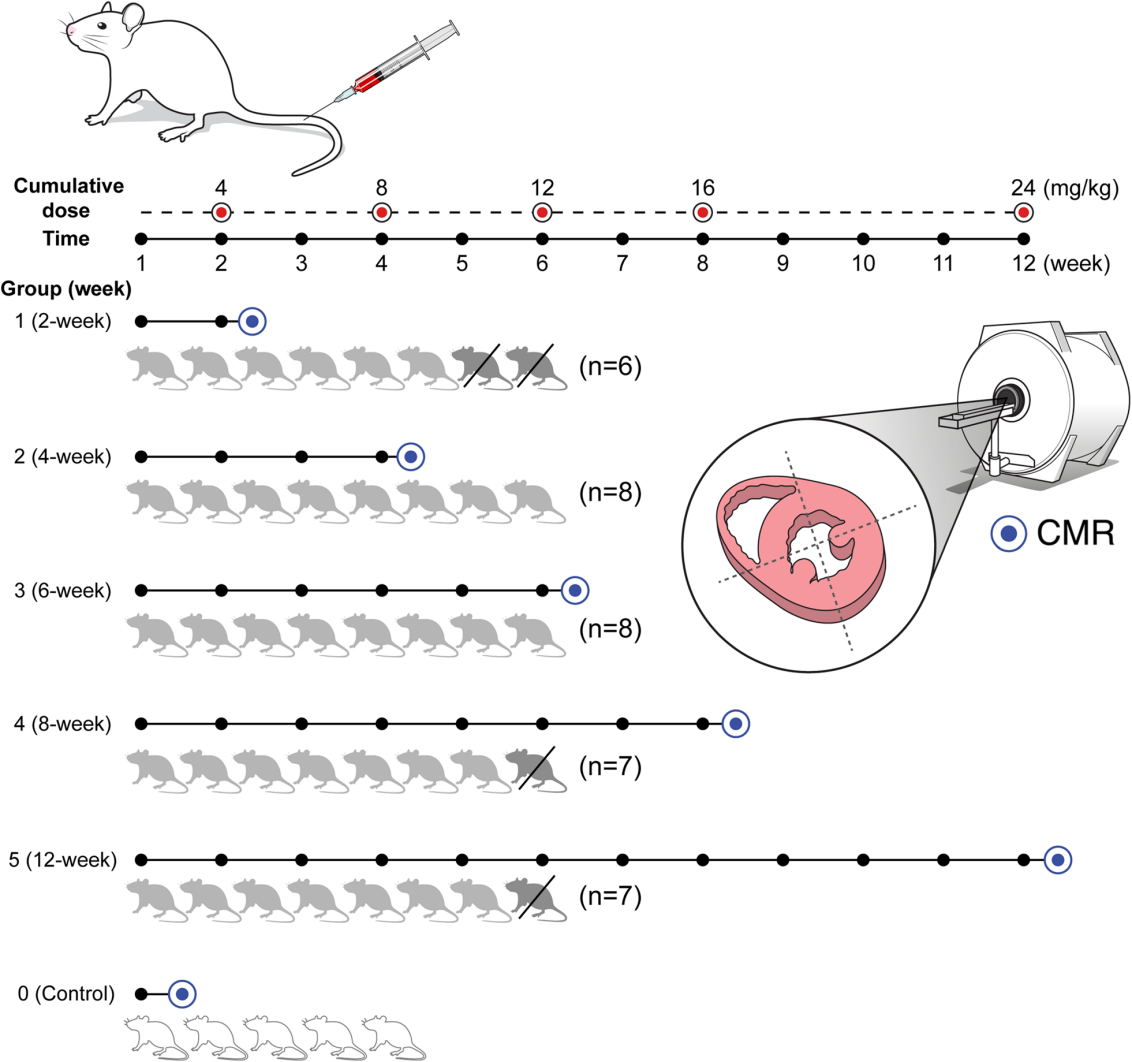
Experimental design and the numbers of subjects in each group. Rats received intravenous doxorubicin (1 mg/kg, twice a week). A total of 40 rats were consecutively enrolled into five groups (eight rats in each group; group 1: 2-week, 2: 4-week, 3: 6-week, 4: 8-week, 5: 12-week). Among them, two rats in group 1 and one each in groups 4 and 5 died during the specific treating period. Five control rats (group 0) that received normal saline intravenously (1 mg/kg, twice a week) were also included for comparison. Cardiovascular magnetic resonance (CMR) scans (blue circles) were performed in the short-axis planes in each group. All rats were sacrificed for histopathologic evaluation immediately after the CMR scan. The association between the CMR parameters and the histopathological changes was assessed

All experiments were approved by our institutional Animal Care and Use Committee and were performed according to the National Institutes of Health guidelines [16]. Five control rats that received normal saline intravenously (1 mg/kg, twice a week) were consecutively included. A total of 40 male Sprague–Dawley rats were also consecutively assigned into five specific doxorubicin treatment periods (Groups 1–5): 2-, 4-, 6-, 8-, and 12-week treatments (cumulative dose: 4, 8, 12, 16, 24 mg/kg, respectively) with eight rats per treatment period (Fig. 1). The sample size estimation was performed using PASS software (version 12, NCSS, Kaysville, Utah, USA) with a two-sample t-test. Based on a previous study [8], we hypothesized that the ECV would be elevated compared to control rats after 3 weeks of treatment. The mean ECV and standard deviation and pooled sample variance from the previous study were used for the sample size calculation. A statistical power of 0.80 and type I error of 0.05 required 7 rats per group. Considering a 10% drop out rate, a total of 40 rats (8 in each group) were included in this study. Doxorubicin-treated rat models were generated by administering doxorubicin (anthracycline agent) to rats (1 mg/kg injection via tail vein, twice a week during the treatment periods) under inhalational anesthesia.

### Cardiovascular magnetic resonance

Rats underwent high field CMR at the end of their specific treatment periods. Immediately before the CMR examination, venous sampling was conducted via the tail vein to determine the hematocrit (Hct) of all rats. Inhalational anesthesia was induced using a mixture of oxygen and isoflurane.

The CMR examination was performed with a 9.4 T scanner (Bruker, Billerica, Massachusetts, USA). The protocol included cine, native T1, and T2, and post-contrast T1 mapping sequences. Cine images in the two- and four-chamber long-axis and short-axis planes were acquired using a fast low-angle shot sequence with electrocardiographic (ECG) gating and the following parameters: echo time/repetition time (TE/TR), 4.0/1.16; flip angle, 15°; 25 phases; section thickness, 2 mm; two signals acquired; acquisition matrix, 128 × 128; and field of view, 50 × 50 mm.

Native and post-contrast T1 mapping images were acquired before and 15 min after the injection of gadoterate meglumine (0.2 mmol/kg body weight, Dotarem; Guerbet, Roissy, France) via the tail vein.

### T1 and T2 mapping sequences

Native T1 mapping was performed with the saturation recovery Look-Locker sequence due to the high heart rate of rats (up to 300–400 bpm) [17]. The detailed pulse diagram of the T1 mapping sequence is in the Additional file 1. This sequence employed a saturation recovery pulse to reset magnetization history at the beginning of acquisition, immediately after ECG triggering. Subsequently, saturation recovery-weighted images were acquired through a series of several heartbeats by prospective ECG-gated cine imaging. The T1 map was obtained by pixel-by-pixel estimation of a mono-exponential function for each cardiac cycle within the acquisition duration. The three-parameter fitting function was: *M* = A − B*exp (− TD/T1)), where M is the signal intensity at saturation recovery delay time (TD), and A and B are scale factors. The parameters were follows: TE/TR, 12/1.35; field of view, 50 × 50 mm; acquisition matrix, 128 × 128; section thickness, 1.5 mm; number of sections, 3; section gap, 3 mm (depending on positions of the basal, mid, and apical planes); flip angle, 8°; acquisition duration, 20 heartbeats; and 10 cardiac phases (depending on the heart rate).

T2 mapping was performed using a spin-echo imaging sequence. ECG and respiratory gating were used to reduce physiological motion artifacts. T2 mapping parameters were as follows: TR, 1500 ms; TE, 16, 26, 36 ms; FOV, 50 × 25 mm; matrix, 128 × 64; spatial resolution, 0.39 × 0.39 mm; excitation slice thickness, 1.5 mm; refocusing slice thickness, 3.0 mm; number of slices, 3. Pulse sequence diagrams of T2 mapping are in the Additional file 1.

### Image analysis

Two expert radiologists (YJH and CHP) with 11 years of experience in cardiovascular image interpretation, who were blinded to the group assignment, analyzed the images independently. All images were analyzed using cvi42 software (Circle Cardiovascular Imaging, Calgary, Alberta, Canada).

### CMR analysis for left ventricular function and mass measurement

LV function, size, and mass were assessed on short-axis cine images. First, the endocardial and epicardial borders of the LV wall were manually delineated and the contours were modified by software on the end-diastolic and end-systolic images. The LV end-diastolic volume and end-systolic volume were measured, and the LVEF (%) was calculated.

### CMR analysis for tissue characterization: T1 and T2 mapping analysis and ECV calculations

**Fig. 2:**
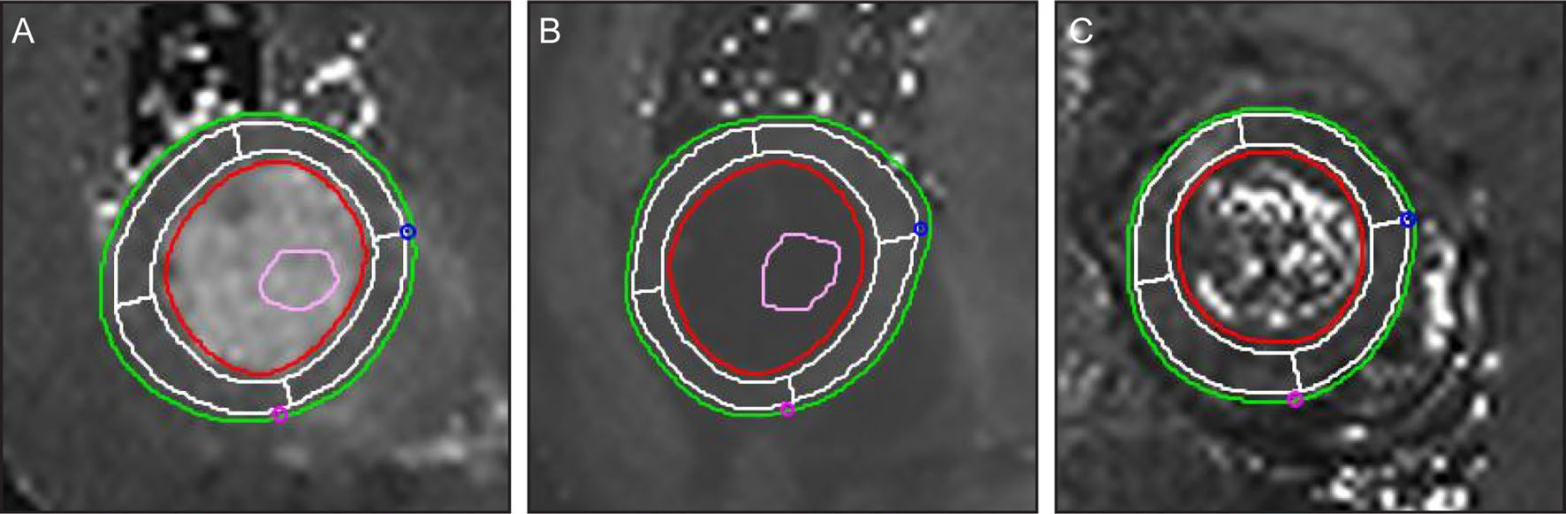
Measurement of the native T1, post-T1, and T2 values in the left ventricular (LV) myocardium. The endocardial (red line) and epicardial borders (green line) of the LV wall were delineated semi-automatically; four segments (i.e. anterior, inferior, septal, and lateral segments) were delineated automatically by applying 15% offset to avoid partial volume artifacts (white lines). A round < 5-mm^2^ region of interest (orange circle) was drawn in the LV cavity on pre-contrast (**a**) and post-contrast T1 (**b**), and T2 (**c**) mapping images. The extracellular volume fraction (ECV) was calculated using the native T1, post-T1, and hematocrit values

Native T1, post-T1, T2 and ECV values were measured at the mid ventricle. On a native T1 image, a post-T1 image obtained at the mid ventricular level in a short-axis view, the endocardial and epicardial borders of the LV wall were drawn and divided into four segments: anterior, inferior, septal, and lateral segments. A 15% offset was applied to avoid partial volume artifact. A round, < 5 mm^**2**^ ROI that avoided the papillary muscle was also drawn in the LV cavity (Fig. 2). The myocardial ECV of each segment was automatically calculated using the Hct value and the native and post*-*contrast T1 values of the LV myocardium and blood cavity using the equation in [18].

### Evaluation of histopathological features in animal models

After undergoing CMR, each rat was euthanized in an unconscious state and placed in a closed CO_2_ gas chamber. Immediately after euthanasia, the heart was removed and fixed in 10% neutral buffered formalin. After 1 week of formalin fixation, a cross section of the whole heart was sampled along the short axis at the mid ventricular level, which was similar to the CMR plane. The tissue sections were processed and embedded in paraffin. Sections with a 4-µm thickness were cut from the paraffin block and stained with hematoxylin and eosin (H&E), Masson’s trichrome, and picrosirius red stain.

Histopathological features including myocyte injury, interstitial fibrosis, inflammation, and edema were evaluated by a pathologist (HSP), blinded to the group assignment and the results of the CMR measurements.

### Quantification of myocyte injury

Myocyte injury was assessed by the presence of intracytoplasmic vacuoles in myocytes. Tissue sections from the LV were quadrisected into septal, anterior, inferior, and lateral segments on a glass slide. The ratio of myocytes with cytoplasmic vacuolization to overall myocytes was evaluated on a microscopic high-power field (HPF) at a magnification of × 200 using a microscope (Olympus Corporation, Tokyo, Japan). The number of cells with vacuolization per 100 cells was counted. Ten high power fields (HPFs) of each LV segment (i.e. anterior, posterior, lateral, and septal segment) were evaluated. In total, 40 HPFs per subject were investigated and the average value was used.

### Image segmentation and quantification of interstitial fibrosis

Interstitial fibrosis was evaluated by determining the collagen proportions in the tissue. First, picrosirius red stained slides were scanned using a digital slide scanner (Pannoramic SCAN, 3DHISTECH Ltd., Budapest, Hungary). Then, whole area at a magnification of × 200 was captured in digitized slides using CaseViewer software (3DHISTECH Ltd.). Ten areas of each LV segment (i.e., anterior, posterior, lateral, and septal segment) were selected and 40 microscopic images per rat were investigated.

The fraction of fibrosis on each histopathologic image was quantified using pixel-based segmentation. Microscopic images in JPEG format were converted from RGB into CIE L*a*b space [19, 20] and segmented into three clusters: collagen fiber, cardiac muscle, and background, using the k-means clustering algorithm [21, 22]. Clustering procedures were internally iterated 100 times in order to maximize segmentation accuracy. The degree of interstitial fibrosis was calculated based on the total pixel number of the collagen fiber cluster. Custom written scripts in Matlab 2020a (MathWorks, Natick, Massachusetts, USA) were used for the quantification steps.

Inflammation and edema were evaluated by H&E and Massons’ trichrome staining. The typical inflammatory cell findings (e.g., cells with segmented nuclei for neutrophils; cells with the perinuclear hop and clock-face chromatin for plasma cells; small dark, round nuclei with scanty cytoplasm for lymphocyte, etc.) were considered as having inflammation. Widening of the interstitial space and pale pink fluid collection and/or surrounding inflammation and cellular reaction (e.g. loose fibrosis) were considered as edema [23]. Interstitial inflammation and edema were graded according to the amount of inflammatory cells present and an observed increase in interstitial space, respectively. The degrees of interstitial inflammation and edema in each segment were graded according to their degree and then scored (0: absent; 1: minimal; 2: mild; 3: moderate; 4: severe). The average value of all segments was determined.

### Statistical analysis

Continuous variables are expressed as means ± standard deviations, and categorical variables are expressed as frequencies or percentages. The Shapiro–Wilk test was performed to evaluate the distribution of the data. Comparisons of baseline characteristics, ventricular functional parameters, T1 mapping values, and the four histopathologic parameters between the groups were performed by using a t-test or ANOVA or Kruskal–Wallis test. Post-hoc analysis was done by Bonferroni correction.

The relationship between the T1 mapping parameters and relevant histopathological changes was examined by a linear regression analysis.

A multiple linear regression model was used to identify independent histopathologic factors for changes in native T1 and ECV using reconstructed models with all variables yielding *P*-values of < 0.05 or a model that excluded the lowest correlated variable in previous univariable analyses. A *P*-value of < 0.05 was considered to be statistically significant. All statistical analyses were performed using SPSS (version 23.0.0, Statistical Package for the Social Sciences, International Business Machines, Inc., Armonk, New York, USA).

## Results

In total, five control rats, six 2-week treated rats (cumulative dose: 4 mg/kg), eight 4-week treated rats (8 mg/kg), eight 6-week treated rats (12 mg/kg), seven 8-week treated rats (16 mg/kg), and seven 12-week treated rats (24 mg/kg) were included in this study. A total of four rats (two in group 1 and one each in groups 4 and 5) were excluded due to their unexpected deaths during their specific treatment periods. CMR was performed after each specific treatment period. Immediately after CMR, rats were sacrificed for histopathologic evaluations. The experimental timeline and the numbers of included and excluded rats, and cumulative dose according to the treatment periods (Groups 1–5) are shown in Fig. 1.

### Physiological and functional data

Compared to control rats, the doxorubicin-treated rats showed a significantly lower Hct (58.8 ± 2.0% vs. 43.3 ± 12.0%, *P* = 0.001), cardiac output (0.15 ± 0.01 l/min vs. 0.12 ± 0.03 l/min, *P* = 0.009), and LVEF (73 ± 4% vs. 65 ± 8%, *P* = 0.03), and a higher LV end-systolic volume (LVESV) (0.18 ± 0.02 ml vs. 0.24 ± 0.05 ml*, P* = 0.05). LV mass and heart rate were not significantly different between the controls and doxorubicin-treated rats. In the subgroup analysis, the LVEF significantly decreased in 12-week treated rats (73 ± 4% vs. 59 ± 9%, *P* = 0.01) (Table 1).

**Table 1:** Physiological and cardiovascular magnetic resonance data for all subjects

	Group 0 Control (n = 5)	Group 1 2-week (n = 6)	Group 2 4-week (n = 8)	Group 3 6-week (n = 7)	Group 4 8-week (n = 7)	Group 5 12-week (n = 7)
Hct (%)	58.8 ± 2.0	41.6 ± 13.0	46.3 ± 10.0	40.5 ± 12.0	43.9 ± 12.0	43.0 ± 14.0
Heart rate (min)	281 ± 30	270 ± 24	280 ± 35	271 ± 28	279 ± 51	292 ± 51
LVEDV (ml)	0.69 ± 0.05	0.73 ± 0.09	0.68 ± 0.09	0.77 ± 0.05	0.69 ± 0.09	0.63 ± 0.01
LVESV (ml)	0.18 ± 0.02	0.20 ± 0.04	0.23 ± 0.03	0.27 ± 0.04	0.24 ± 0.06	0.26 ± 0.07
LVSV (ml)	0.51 ± 0.06	0.52 ± 0.06	0.46 ± 0.10	0.50 ± 0.05	0.45 ± 0.07	0.37 ± 0.10
CO (l/min)	0.15 ± 0.01	0.14 ± 0.01	0.13 ± 0.03	0.14 ± 0.03	0.13 ± 0.03	0.10 ± 0.03^*^
LV mass (g)	0.66 ± 0.08	0.69 ± 0.09	0.67 ± 0.14	0.74 ± 0.11	0.64 ± 0.08	0.61 ± 0.06
LVEF(%)	73 ± 4	72 ± 3	66 ± 8	65 ± 5	65 ± 7	59 ± 9^*^
Native T1 (ms)	1148 ± 58	1191 ± 119	1278 ± 49	1320 ± 56^*^	1327 ± 82^*^	1316 ± 69^*^
T2 (ms)	10.3 ± 1	12.2 ± 1	11.4 ± 1	16.3 ± 2^*^	17.3 ± 5	14.0 ± 2
Partition coefficient	0.36	0.28	0.32	0.35	0.38	0.43^†^
ECV (%)	14.3 ± 1	15.7 ± 1	17.1 ± 2	20.3 ± 3^*^	21.2 ± 4^*^	23.2 ± 4^*^

Values are expressed as mean or mean ± SD

*CO* cardiac output; *ECV* extracellular volume fraction; *Hct* hematocrit; *LVEDV* left ventricular end-diastolic volume; *LVEF* left ventricular ejection fraction; *LVESV* left ventricular end-systolic volume; *LVSV* left ventricular stroke volume, min; minute

*Control vs. Group subjects, *P* < 0.05

^†^Group 1 vs Group 5, *P* < 0.05

### T1 and T2 mapping data

In doxorubicin-treated rats, the native T1, ECV, and T2 values were higher than in the controls (controls vs. doxorubicin-treated rats: 1148 ± 58 ms, 14.3 ± 1%, 10.3 ± 1 ms vs. 1292 ± 84 ms, 19.7 ± 4.0%, 14.6 ± 3 ms; *P* = 0.001, < 0.001, < 0.05, respectively).

In the subgroup analysis, a comparison of each group revealed that the native T1 and ECV values were significantly higher in the 6-week treatment (1320 ± 56 ms, 20.3 ± 3.0%, *P* = 0.005, < 0.05, respectively), 8-week treatment (1327 ± 82 ms, 21.2 ± 4.0%, *P* = 0.003, 0.01, respectively), and 12-week treatment rats (1316 ± 69 ms, 23.2 ± 4.0%, *P* = 0.006, 0.001, respectively) than in the controls (1148 ± 58 ms, 14.3 ± 1.0%). Although native T1 and ECV values in rats treated for longer periods were higher than in those treated for shorter periods, there were no significant differences amongst treatment periods. T2 values were significantly higher in the 6-week treated rats than in control rats (16.3 ± 2.0 ms vs. 10.3 ± 1.0, *P* = 0.03) (Table 1).

### Histopathologic data

Histopathological changes were noted in the doxorubicin-treated rats. The main histopathological findings were vacuolar changes in myocytes, interstitial edema, inflammation, and fibrosis.

**Fig. 3:**
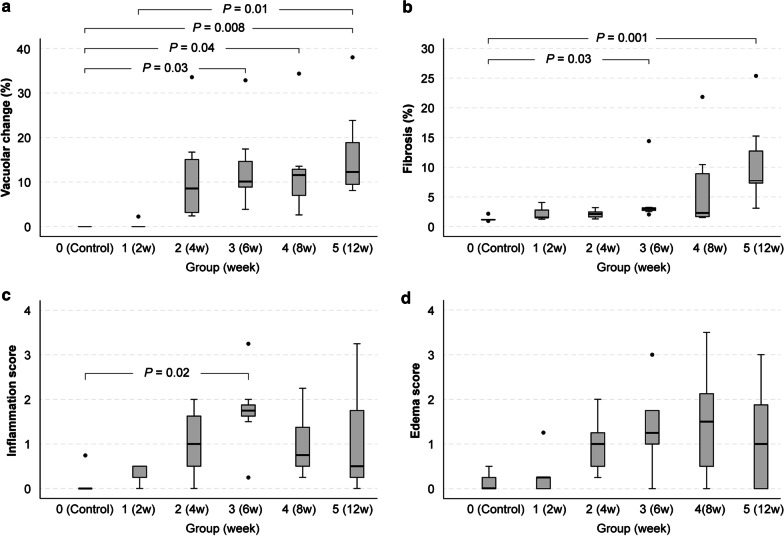
Histopathological data for all subjects. The degree of myocardial injury (vacuolar change, **a**), fibrosis (**b**), score of myocardial inflammation (**c**), scores of interstitial edema (**d**) in controls (group 0), and groups 1–5. Groups 1–5 indicate 2-week, 4-week, 6-week, 8-week, and 12-week treated subjects. Group 1 (2 week), 2 (4 week), 3 (6 week), 4 (8 week), and 5 (12 week) indicate subjects undergoing 2, 4, 6, 8, and 12 weeks of treatment. **a** The mean vacuolar changes (range) in groups 1–5 were 0.5% (0–2.3%), 11.2% (2.4–33.6%), 13.4% (3.9–32.9%), 12.7% (2.6–35.0%) and 16.5% (8.1–38.0%), respectively. The mean values of vacuolar change were significantly higher in group 3 (*P* = 0.03), group 4 (*P* = 0.04), and group 5 subjects (*P* = 0.008) than in the control subjects (0%, 0–0%). The mean value of group 5 was also significantly higher than group 1 (*P* = 0.01). **b** The mean fibrosis (range) in groups 1–5 was 2.2% (1.3–4.1%), 2.1% (1.3–3.2%), 4.5% (2.1–14.4%), 6.7% (1.5–21.8%) and 11. 0% (3.1–25.4%), respectively. The mean values of fibrosis were significantly higher in groups 3 (*P* = 0.03) and 5 (*P* = 0.001) than in the control rats (1.2%, 1.0–1.6%). **c** The median scores of inflammation (range) of groups 1–5 were 0.25 (0–0.50), 1.00 (0–2.00), 1.75 (0.25–3.25), 0.75 (0.25–2.25), and 0.50 (0–3.25), respectively. There was a significant difference only between group 3 and control subjects (*P* = 0.02). **d** The median scores of edema (range) of groups 1–5 were 0.25 (0–1.25), 1.00 (0.25–2.00), 1.25 (0–3.00), 1.5 (0–3.50), and 1.00 (0–3.00), respectively. There was no significant difference between groups

**Fig. 4:**
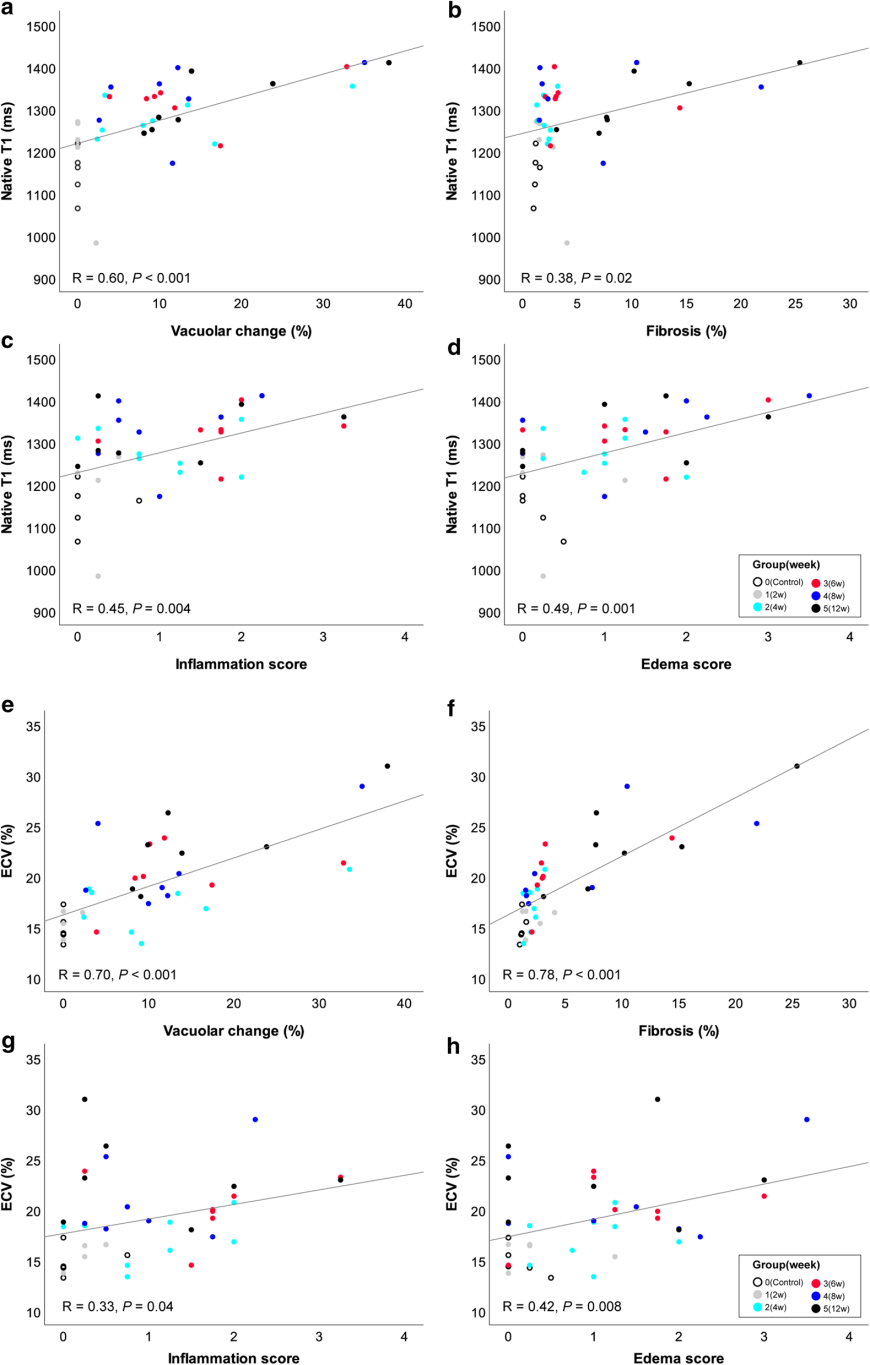
Univariable regression analysis between native T1 or ECV and histopathological findings. Groups 1 (2 week), gray circle; 2 (4 week), emerald circle; 3 (6 week), red circle; 4 (8 week), blue circle; and 5 (12 week), black circle indicates the subjects undergoing 2, 4, 6, 8, and 12 weeks of treatment. Group 0 (control), open circle indicates the control subjects. **a–d** The native T1 value showed significant association with the vacuolar change (**a**, R = 0.60, *P* < 0.001), fibrosis (**b**, R = 0.38, *P* = 0.02), inflammation score (**c**, R = 0.45, *P* = 0.004), and myocardial edema score (**d**, R = 0.49, *P* = 0.001). **e–h** ECV value showed significant association with the vacuolar change (**e**, R = 0.70, *P* < 0.001), fibrosis (**f**, R = 0.78, *P* < 0.001), inflammation score (**g**, R = 0.33, *P* = 0.04), and myocardial edema score (**h**, R = 0.42, *P* = 0.008)

The mean vacuolar changes (range) in groups 1–5 were 0.5% (0–2.3%), 11.2% (2.4–33.6%), 13.4% (3.9–32.9%), 12.7% (2.6–35.0%) and 16.5% (8.1–38.0%), respectively. The vascular changes in groups 3 (*P* = 0.03), 4 (*P* = 0.04), and 5 (*P* = 0.008) were significantly higher than in the control rats (0%, 0–0%). The change in group 5 was also significantly higher than group 1 (*P* = 0.01). The mean fibrosis (range) in groups 1–5 were 2.2% (1.3–4.1%), 2.1% (1.3–3.2%), 4.5% (2.1–14.4%), 6.7% (1.5–21.8%) and 11.0% (3.1–25.4%), respectively. The mean fibrosis in groups 3 (*P* = 0.03) and 5 (*P* = 0.001) were significantly higher than in the control rats (1.2%, 1.0–1.6%). The median scores of inflammation (range) of groups 1–5 were 0.25 (0–0.50), 1.00 (0–2.00), 1.75 (0.25–3.25), 0.75 (0.25–2.25), and 0.50 (0–3.25), respectively. The only significant difference was noted between group 3 and control rats (*P* = 0.02). Lastly, the median scores of edema (range) of groups 1–5 were 0.25 (0–1.25), 1.00 (0.25–2.00), 1.25 (0–3.00), 1.5 (0–3.50), and 1.00 (0–3.00). There was no significant difference between groups (Fig. 3). In the univariable regression analysis, native T1 value showed significant association with the vacuolar change (R = 0.60, *P* < 0.001), myocardial edema score (R = 0.49, *P* = 0.001), inflammation score (R = 0.45, *P* = 0.004), and fibrosis (R = 0.38, *P* = 0.02). ECV showed significant association with fibrosis (R = 0.78, *P* < 0.001), vacuolar change (R = 0.70, *P* < 0.001), myocardial edema (R = 0.42, *P* = 0.008), and inflammation (R = 0.33, *P* = 0.04) (Fig. 4). The highest correlated parameters were vacuolar change for a native T1, and fibrosis for ECV (R = 0.60, 0.78, respectively).

There was no significant associated with a native T1 in a model with all four histopathologic parameters in the multivariable regression analysis. When the lowest correlated parameter, i.e., fibrosis (R = 0.38), was not included in the model, vacuolar change was a significant independent factor for changes in the native T1 value (*P* = 0.01) with an adjusted R^2^ of 0.34. However, there was no improvement in adjusted R^2^ compared to the vacuolar change for T1 value in the univariable analysis (R^2^ of 0.35). Vacuolar change and fibrosis were significant factors for changes in ECV (*P* = 0.006, < 0.001, respectively) with an adjusted R^2^ of 0.74. By adding other histopathological parameters (i.e., inflammation and edema scores), there was an improvement of R^2^ over vacuolar change (R^2^ of 0.48) or fibrosis (R^2^ of 0.60) for ECV in univariable analysis (Table 2). When the lowest correlated parameter, i.e., inflammation (R = 0.33), was not included in the model, vacuolar change and fibrosis were still significant factors for changes in ECV (*P* = 0.004, < 0.001, respectively) with an adjusted R^2^ of 0.73.

**Table 2:** Multiple linear regression analysis for the native T1 value and ECV

	Regression coefficient	Standard error	*P*-value	VIF	R^2^	Adjusted R^2^
Native T1						
Vacuolar change	4.7	1.8	**0.01**	2.2	0.39	0.34
Inflammation	21.0	18.2	0.26	1.8		
Edema	− 1.7	21.8	0.94	2.9		
ECV						
Vacuolar change	0.17	0.06	**0.006**	3.0	0.76	0.74
Fibrosis	0.44	0.07	** < 0.001**	1.4		
Inflammation	0.60	0.52	0.26	1.8		
Edema	− 0.39	0.64	0.55	3.0		

In each model, all independent variables had no multicollinearity (VIF < 8)

*ECV* extracellular volume fraction

Bold values indicate statistical significance with *P* < 0.05

## Discussion

The purpose of this study was to determine the surrogate T1 and T2 mapping parameters acquired using ultrahigh-field T1 CMR against histopathologic features in cardiotoxicity. We created rat models of cardiotoxicity and performed CMR at different treatment time points and immediately sacrificed the animals to compare changes in CMR and tissue findings. We thoroughly reviewed the histopathologic changes that occurred in doxorubicin-treated and control rats. We also investigated pathological factors that are significantly associated with changes in T1 and ECV.

**Fig. 5:**
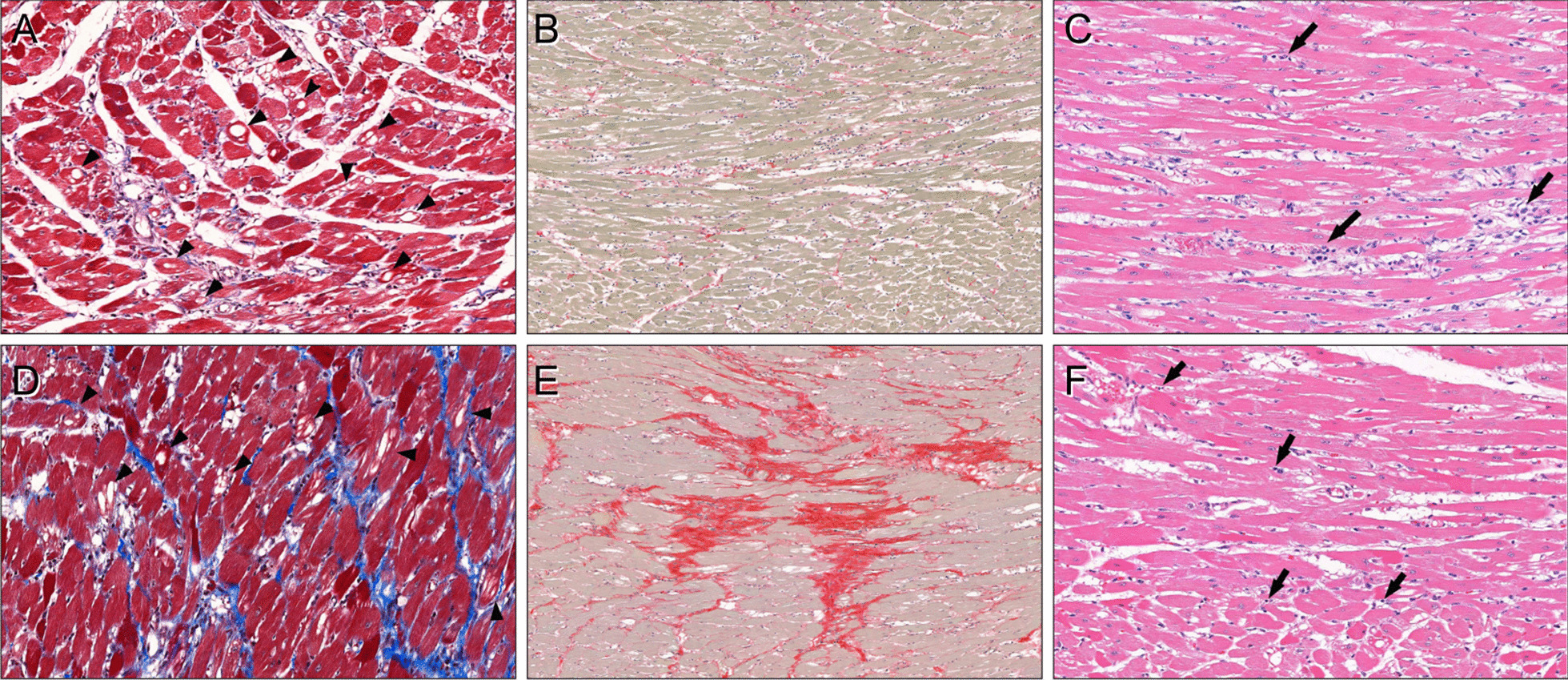
CMR data and histopathological findings of doxorubicin-treated rats. **a–c** For a representative rat at 6 weeks of doxorubicin treatment (group 3) with a left ventricular ejection fraction (LVEF) of 65%, global native T1 of 1401 ms, and ECV of 21.3%, there were marked vacuolar changes (arrowheads) of the myocytes with a mean value of 32.9% (**a**, Masson’s trichrome stain, × 200). Interstitial fibrosis was 3.0% (**b**, picrosirius red stain, × 100). There were interstitial edema (median score: 2) and infiltration of lymphohistiocytic aggregates (arrows, median score: 2) around the injured myofibers (**c**, hematoxylin, and eosin stain, × 200). **d–f** For a representative rat at 12 weeks of doxorubicin treatment (group 5) with a LVEF of 67%, global native T1 of 1410 ms, and ECV of 28.9%, there were multifocal vacuolar degenerations in the myocytes (arrowheads) with a mean vacuolar change of 38.0% (**d**, Masson’s trichrome stain, × 200). Interstitial fibrosis was severe, with a mean value of 25.4% (**e**, picrosirius red stain, × 100). Interstitial edema (median score: 1.75) and scattered lymphocytic infiltration (arrows, median score: 0.25) were also present (**f**, hematoxylin and eosin stain, × 200)

Our data demonstrate that the anthracycline agent caused various histopathologic changes, which included vacuolar changes in myocytes (i.e. myocyte injury), interstitial edema, inflammation, and fibrosis. Additionally, most rats exhibited elevated native T1 and ECV values with concomitant histopathological changes (Fig. 5). Mild vacuolar change was noted early in treatment (4 weeks) and significantly changed at 6 weeks. Mild interstitial fibrosis was significant change at 6 weeks. Although there was no significant difference between doxorubicin-treated groups, vacuolar change and fibrosis were more severe in rats treated for longer periods. However, myocardial inflammation and edema scores appeared to have variations between each group. T1 and ECV values were elevated in doxorubicin-treated rats, and both significantly changed at 6 weeks. However, LVEF did not change significantly until 12 weeks of treatment.

Among histopathological findings, the vacuolar change showed the highest correlation with native T1 (R = 0.60), and fibrosis showed highest correlation with ECV (R = 0.78). In the multivariable analysis model, vacuolar change was a significant factor for native T1 changes, and vacuolar change and interstitial fibrosis were significant factors for ECV changes, which suggests they could be valid CMR markers for tissue changes in cardiotoxicity.

Currently, most guidelines use LVEF as a diagnostic indicator for chemotherapy-induced cardiotoxicity. However, the main mechanism of cardiotoxicity is microstructural injury to the myocardium due to direct effects of toxicity [24]. Factors that impair LV systolic function do so by either compromising the function of the myofibrils, or by injuring or destroying cardiomyocytes [1, 25]. The relationship between microstructural injury to the myocardium and the course of contractile dysfunction remains poorly understood.

A previous study suggested that chemotherapy-induced cardiotoxicity is considered to be a continuum that begins with subclinical myocardial cell injury and leads to an early asymptomatic decline in the LVEF that can develop into symptomatic heart failure [5]. At the tissue level, early anthracycline toxicity has been associated with myocardial inflammation [26–29], vacuolization [15, 30, 31], and cell swelling/edema [26, 31, 32]. Later stages of toxicity are associated with myocardial fibrosis [26, 33, 34]. Our results also supported the observation that myocardial tissue changes seem to occur at an earlier stage. Therefore, LVEF measurement, which does not allow for early preventive strategies [7], is a method for detecting myocardial damage only after irreversible changes have already occurred in the course of a disease. An effective method for the early detection of myocardial tissue changes is needed. Therefore, we need an effective diagnostic tool that can identify the changes in these tissues at the early stages of the disease, and T1, T2-mapping CMR may be a promising tool. CMR is the gold standard technique that allows accurate anatomical and functional evaluation [35]. Moreover, myocardial T1 mapping has become a mainstream sequence in imaging various cardiomyopathies, given its quantitative ability for tissue characterization. This technique now enables us to assess myocardial changes such as myocardial edema [36], inflammation [36], or fibrosis, which are representative features of anthracycline-induced cardiotoxicity, by quantifying extracellular matrix expansion using ECV measurement [18, 37].

There are a few animal studies that evaluate CMR in animal models of cardiotoxicity.

In addition to other studies, we tried to find surrogate T1 mapping imaging markers against various histopathologic features in anthracycline-induced cardiotoxicity.

Lightfoot et al. showed the potential of qualitative imaging biomarkers as predictors of cardiotoxicity in rats. An early increase in signal intensity can predict the subsequent occurrence of cardiotoxicity [38]. Fahad et al. [14] performed serial CMR scans in mice. They reported an early increase in native T1 and T2 values due to myocardial edema and a subacute increase in ECV due to myocardial fibrosis. Both early edema and later fibrosis were predictors of later mortality. Galan-Arriolar et al. [9] examined the effects of cardiotoxicity in pigs with intracoronary doxorubicin administration. In their study, T2 values increased in early stages of cardiotoxicity without changes in T1 or ECV values. In our study, we focused on imaging and histopathologic changes in the earlier period and attempted to find which histopathologic changes were factors for changes in T1, T2, and ECV values. In our results, myocardial edema and inflammation were not noted in all treated rats, but were noted in some. Changes in T2 values are probably due to myocardial edema or inflammation. Changes in T1 and ECV values were noted in earlier-treated animals with histologic evidence of cytoplasmic vacuolization of myocytes.

Similar to the previous results, ECV and T1 values significantly changed in the earlier period more so than LVEF, and these changes were well-correlated with concomitant histopathologic changes. Unlike previous studies, native T1 changed in the early period, which was found to be due to vacuolar change, myocardial inflammation, and edema. As demonstrated in our study, anthracyclines are directly toxic to the myocyte, and induce myocyte injury as well as inflammation, interstitial edema, and fibrosis.

The myocardial damage and other pathologic changes induced by anthracyclines at subclinical doses could not be detected by LVEF measurement in many models, although it can be quantitatively evaluated using noninvasive native T1-and T2- mapping CMR.

### Limitations

There are some limitations to this study. First, T2 mapping sequences were conducted in some rats (n = 24). It is necessary to find histopathologic factors for T2 changes in a larger sample size, as was done for native T1 and ECV. Second, LGE imaging was not possible due to the scan time limitation. The LGE pattern of chemotherapy-induced cardiotoxicity should be verified in future studies. Third, in the histopathologic analysis, inflammation and edema scores were qualitative and based on the assessment of a single pathologist. Additionally, special stains for the quantification of inflammatory cells were not conducted. Fourth, the experiment was performed using sample size calculation based on the previous study, however, given the circumstances, the control group has a smaller sample size. Lastly, in our study the LVEF decrease was only evident in the 12 week-rats, far later than the previous studies which could be an artifact of the wide variability between rats in response to the chemotherapy. Additionally, baseline data was not acquired before doxorubicin treatment. Further research is required to investigate the variability between the chemotherapy and the role of quantitative imaging parameters including T1 and T2 mapping sequences in diagnosing chemotherapy-induced cardiotoxicity in human subjects in a clinical setting.

## Conclusion

In conclusion, our findings in doxorubicin-treated rats suggest that T1 and T2 mapping CMR-based assessment of cardiotoxicity may have potential as a non-invasive tool for the evaluation of subclinical myocardial changes.

## Supplementary Information

**Additional file 1: **Pulse sequence diagrams for T1 and T2 mapping.

## Data Availability

All data generated or analyzed during this study are included in this published article and its supplementary information files.

## Abbreviations

CMR: Cardiovascular magnetic resonance
CO: Cardiac output
ECG: Electrocardiogram
ECV: Extracellular volume
H&E: Hematoxylin and eosin
Hct: Hematocrit
HPF: High-power field
LV: Left ventricle/left ventricular
LVEDV: Left ventricular end-diastolic volume
LVEF: Left ventricular ejection fraction
LVESV: Left ventricular end-systolic volume
LVSV: Left ventricular stroke volume
ROI: Region of interest
TD: Delay time
TE: Echo time
TR: Repetition time
