# Supplementary material for: Ultrahigh-field cardiovascular magnetic resonance T1 and T2 mapping for the assessment of anthracycline-induced cardiotoxicity in rat models: validation against histopathologic changes
Source: J Cardiovasc Magn Reson. 2021 Jun 17;23:76. doi: 10.1186/s12968-021-00767-8 (PMC8210390; doi:10.1186/s12968-021-00767-8)

Pulse sequence diagram for T1 mapping

**ECG**

**Saturation Recovery**

**Look-Locker**

**Net**

**Magnetization**

**Acquisition Duration (AD)**

**Adiabatic Saturation Recovery Pulse**

**CINE imaging**

…

…

…

Pulse sequence diagram for T2 mapping


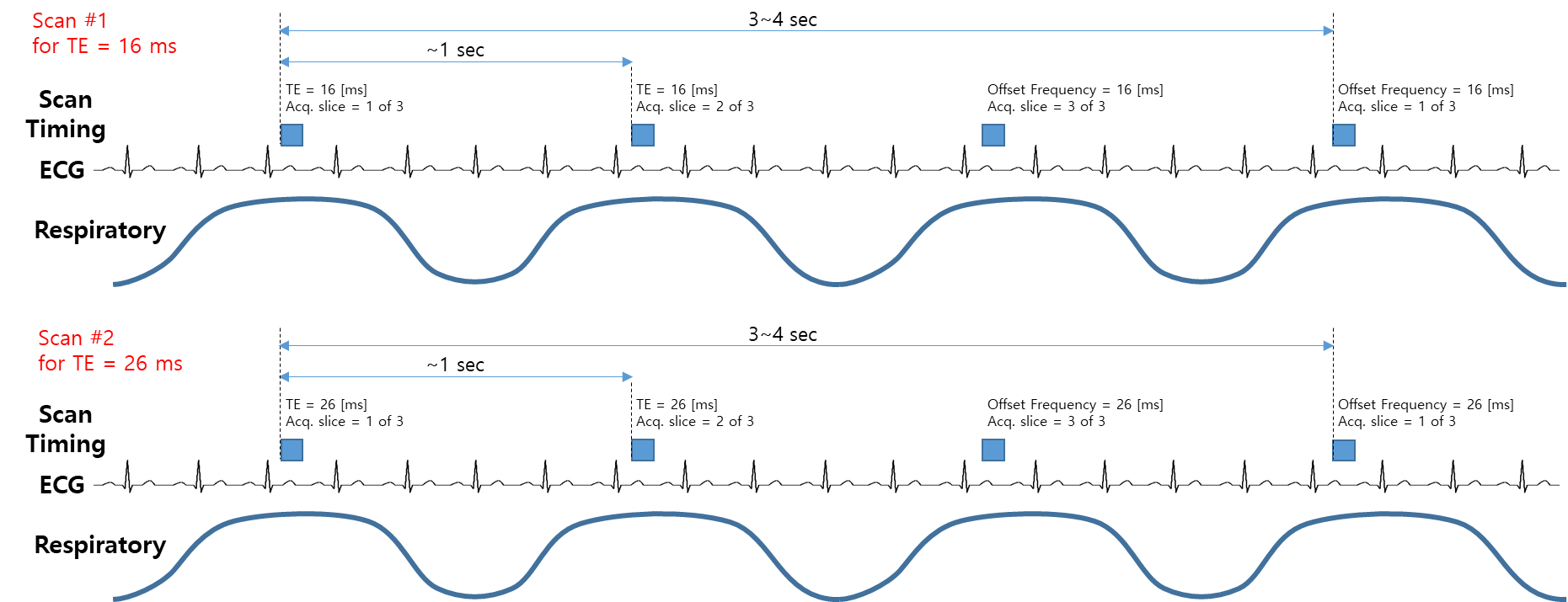

Supplement: Supplementary file 1 — Additional file 1: Pulse sequence diagrams for T1 and T2 mapping. [file 12968_2021_767_MOESM1_ESM.docx]
